# Publisher’s Note: Journal of *Craniomaxillofacial Trauma and Reconstruction* (*CMTR*)

**DOI:** 10.3390/cmtr18010002

**Published:** 2025-01-02

**Authors:** Peter Roth

**Affiliations:** MDPI, St. Alban-Anlage 66, 4052 Basel, Switzerland; roth@mdpi.com

We are excited to welcome the Journal of *Craniomaxillofacial Trauma and Reconstruction* (*CMTR*) to the MDPI family. This new partnership represents a significant milestone in the journal’s evolution, and we are committed to supporting *CMTR*’s mission to advance research in craniomaxillofacial trauma and reconstruction through high-quality, accessible publications.

At MDPI, we understand that no two journals are the same. Each academic community has unique needs, goals, and challenges. That is why we believe flexibility is paramount in publishing. We take pride in our ability to adapt our workflows and services to the specific requirements of our partners. For *CMTR*, this means offering personalized support that aligns with AO CMF’s global vision, while ensuring that the journal continues to serve its readership with excellence and precision.

This adaptability extends to our robust Open Access platform, which ensures that *CMTR*’s cutting-edge research will be freely accessible to a global audience, reaching the clinicians, researchers, and patients who can benefit from it most. By providing this level of flexibility, MDPI empowers journals like *CMTR* to maximize their impact and contribute to advancing the field of craniomaxillofacial surgery.

We are excited about the future of *CMTR* under this new partnership and look forward to working closely with the Editorial Team, AO CMF, and the broader community. Together, we will continue to push the boundaries of research and ensure that *CMTR* remains a vital resource for advancing patient care and outcomes in this critical field of medicine.

Sincerely,

Peter Roth

Head of Publishing MDPI

